# Diffusion Tensor Imaging in Patients with Glioblastoma Multiforme Using the Supertoroidal Model

**DOI:** 10.1371/journal.pone.0146693

**Published:** 2016-01-13

**Authors:** Choukri Mekkaoui, Philippe Metellus, William J. Kostis, Roberto Martuzzi, Fabricio R. Pereira, Jean-Paul Beregi, Timothy G. Reese, Todd R. Constable, Marcel P. Jackowski

**Affiliations:** 1 Department of Radiology, Massachusetts General Hospital, Harvard Medical School, Athinoula A. Martinos Center for Biomedical Imaging, Boston, MA, United States of America; 2 Department of Neurosurgery, Hôpital de la Timone Adultes Marseille, Marseille, Bouches-du-Rhône, France; 3 Rutgers Robert Wood Johnson Medical School, New Brunswick, NJ, United States of America; 4 Laboratory of Cognitive Neuroscience, Brain Mind Institute, School of Life Sciences, Ecole Polytechnique Fédérale de Lausanne, Lausanne, Switzerland; 5 Department of Radiology, University Hospital Center of Nîmes and Research Team EA 2415, Nîmes, Gard, France; 6 Department of Diagnostic Radiology, Yale University School of Medicine, Magnetic Resonance Research Center, New Haven, CT, United States of America; 7 Department of Computer Science, Institute of Mathematics and Statistics, University of São Paulo, São Paulo, Brazil; University of Queensland, AUSTRALIA

## Abstract

**Purpose:**

Diffusion Tensor Imaging (DTI) is a powerful imaging technique that has led to improvements in the diagnosis and prognosis of cerebral lesions and neurosurgical guidance for tumor resection. Traditional tensor modeling, however, has difficulties in differentiating tumor-infiltrated regions and peritumoral edema. Here, we describe the supertoroidal model, which incorporates an increase in surface genus and a continuum of toroidal shapes to improve upon the characterization of Glioblastoma multiforme (GBM).

**Materials and Methods:**

DTI brain datasets of 18 individuals with GBM and 18 normal subjects were acquired using a 3T scanner. A supertoroidal model of the diffusion tensor and two new diffusion tensor invariants, one to evaluate diffusivity, the toroidal volume (TV), and one to evaluate anisotropy, the toroidal curvature (TC), were applied and evaluated in the characterization of GBM brain tumors. TV and TC were compared with the mean diffusivity (MD) and fractional anisotropy (FA) indices inside the tumor, surrounding edema, as well as contralateral to the lesions, in the white matter (WM) and gray matter (GM).

**Results:**

The supertoroidal model enhanced the borders between tumors and surrounding structures, refined the boundaries between WM and GM, and revealed the heterogeneity inherent to tumor-infiltrated tissue. Both MD and TV demonstrated high intensities in the tumor, with lower values in the surrounding edema, which in turn were higher than those of unaffected brain parenchyma. Both TC and FA were effective in revealing the structural degradation of WM tracts.

**Conclusions:**

Our findings indicate that the supertoroidal model enables effective tensor visualization as well as quantitative scalar maps that improve the understanding of the underlying tissue structure properties. Hence, this approach has the potential to enhance diagnosis, preoperative planning, and intraoperative image guidance during surgical management of brain lesions.

## Introduction

Using tensor descriptions of water diffusion, Diffusion Tensor Imaging (DTI) enables the characterization of normal tissue structures as well as alterations due to pathological processes [1–3]. The structural and connectivity information conveyed by DTI has made this technique a powerful tool in neurology and neuro-oncology, leading to an improvement in the diagnosis and prognosis of cerebral lesions, preoperative planning, as well as in neurosurgical guidance for tumor resection [4–9].

One of the fundamental difficulties in interpreting diffusion tensor data lies in its multidimensional and multivariate nature. Furthermore, diffusion tensors can be classified as isotropic or anisotropic. Isotropic tensors have no preferential diffusion direction, while anisotropic tensors have one (orthotropic tensor) or two (transversely isotropic tensor) preferential diffusion directions [10]. This necessitates the use of a tensor representation that can portray this multivariate information in an intuitive manner. Classically, the diffusion tensor has been represented by ellipsoids scaled by the magnitudes of the principal diffusivities and oriented by their principal directions. However, visual ambiguity can confound how the medium and minor eigenvectors are oriented in space and obscure the differences in their magnitudes. Consequently, ellipsoidal fields must be viewed from several perspectives for a comprehensive understanding of the underlying biological architecture. In the human brain, this information is important in the assessment of fascicular organization and connectivity as well as of myelin sheath and cell membrane integrity [11].

To overcome some of the visual ambiguity of the ellipsoidal model, Kindlmann and Ennis devised a tensor representation using superquadric glyphs, which improved upon the ability to distinguish myofiber orientation and structure [12–14]. Superquadric functions are parameterized in order to represent a continuum of convex shapes ranging from discs and spheres to cubes and cylinders. They also provide visual cues (e.g. sharp edges) to highlight distinct fiber orientations. As an example, when compared to ellipsoids, superquadrics improve visualization of transmural myocardial fiber arrangement and tissue structure [12], but depending on the viewing perspective, they may suffer from similar visual ambiguities. Therefore, superquadrics do not always provide effective visualization of different eigenvalue configurations.

In order to analyze and interpret information from DTI data for each voxel, multivariate tensor data are typically reduced to scalar maps through the use of geometric-based tensor invariants and other scalar tensor contractions [10, 15–19]. Derived from the ellipsoidal model, the most commonly used invariants to measure diffusivity and anisotropy are the mean diffusivity (MD) and the fractional anisotropy (FA), respectively.

In the study of glioblastoma multiforme (GBM), Bauer *et al*. used FA, MD, and relative cerebral blood volume (rCBV) to discriminate solitary brain metastasis from GBM [20]. Roldan-Valadez *et al*. found that among 11 DTI-derived tensor metrics, only axial diffusivity, spherical isotropy, and linear anisotropy were selected in a multivariate discriminant model for differentiating brains with and without GBM [21].

In this work, we describe a toroid-based representation, the supertoroid, in an effort to improve upon the ellipsoidal and superquadric glyphs. The supertoroidal tensor representation incorporates the increase in genus inherent to toroids and provides a continuum of shapes that fully encode the local dyadic diffusion tensor [22–24]. In addition, we derive two diffusion tensor invariants with which to measure diffusivity, the toroidal volume (TV), and anisotropy, the toroidal curvature (TC). This approach is then employed and evaluated in the characterization of glioblastoma multiforme (GBM).

## Materials and Methods

### Study subjects

Eighteen subjects with the diagnosis of glioblastoma multiforme (GBM) and eighteen healthy volunteers were selected for this study. The study was approved by the Yale University Human Investigation Committee and written informed consent was obtained from each participant.

### Diffusion tensor imaging (DTI)

DTI brain datasets were acquired using a 3T Siemens Trio whole-body scanner (MAGNETOM Trio, Siemens Healthcare, Erlangen, Germany) with a standard head coil used for signal transmission and reception. A diffusion-weighted single-shot EPI sequence with a gradient set containing 32 directions and two b-values, 0 s/mm^2^ (T2-weighted) and 800 s/mm^2^, was utilized [25–27]. Other parameters were: FOV = 320×320 mm^2^, matrix size = 128×128, in-plane resolution = 2.5 mm, thickness = 3.3 mm, 40 slices, TR = 4700 ms, TE = 76 ms, number of averages = 3, totaling 7.5 minutes of acquisition time. Once all DTI datasets were acquired, motion and distortion correction was performed using both linear and nonlinear registration techniques [28]. Diffusion tensor fields were then computed and the tensor glyphs generated with a custom-built software package.

### Supertoroidal model

As with superquadrics [14], the supertoroidal model provides a continuum of shapes that fully encodes the diffusion eigensystem described by the local dyadic diffusion tensor [24]. The supertoroidal glyph is a function of the geometric shape metrics [29] *C*_*L*_ = (*λ*_1_ − *λ*_2_)/(*λ*_1_ + *λ*_2_ + *λ*_3_), *C*_*P*_ = 2(*λ*_2_ − *λ*_3_)/(*λ*_1_ + *λ*_2_ + *λ*_3_), and *C*_*S*_ = 3*λ*_3_/(*λ*_1_ + *λ*_2_ + *λ*_3_) and is parameterized as follows:

$$C_{S}\geq C_{P}\to ℑ\left(\theta ,\phi \right)=\left(\begin{array}{c} cos^{\eta_{1}}\theta \left\{\left(C_{L}+C_{P}\right)+C_{S}cos^{\eta_{2}}\phi \right\}\\ sin^{\eta_{1}}\theta \left\{\left(C_{L}+C_{P}\right)+C_{S}cos^{\eta_{2}}\phi \right\}\\ sin^{\eta_{2}}\phi \end{array}\right),$$ (1)

$$C_{S}<C_{P}\to ℑ\left(\theta ,\phi \right)=\left(\begin{array}{c} cos^{\eta_{1}}\theta \left\{C_{S}+\left(C_{L}+C_{P}\right)cos^{\eta_{2}}\phi \right\}\\ sin^{\eta_{1}}\theta \left\{C_{S}+\left(C_{L}+C_{P}\right)cos^{\eta_{2}}\phi \right\}\\ sin^{\eta_{2}}\phi \end{array}\right),$$ (2)

where ℑ, the parameterized glyph surface, is a function of both azimuthal *θ* ∈ [0, 2*π*] and polar *ϕ* ∈ [0, 2*π*] coordinates. The parameters $\eta_{1}={\left(1-C_{P}\right)}^{\gamma_{1}}$ and $\eta_{2}={\left(1-C_{P}\right)}^{\gamma_{2}}$ enable a smooth transition between supertoroidal shapes. The role of *γ*_1_ is to control the sharpness of the shape in the medium and minor diffusion directions. The parameter *γ*_2_ controls the shape along the orthogonal axis, which is aligned with the major direction of diffusion. The parameters *γ*_1_ and *γ*_2_ were chosen to have values 4 and 0.5, respectively, for the shapes used in this study. These values were chosen to maximize the visual effectiveness of the eigensystem. Note that while the parameterization of the supertoroid results in a continuum of genus 1 shapes, it can produce both genus 1 and genus 0 glyphs. For instance, in an isotropic region, when no preferential diffusion direction exists, it will resemble a genus 0 glyph (will have no hole).

**Fig 1:**
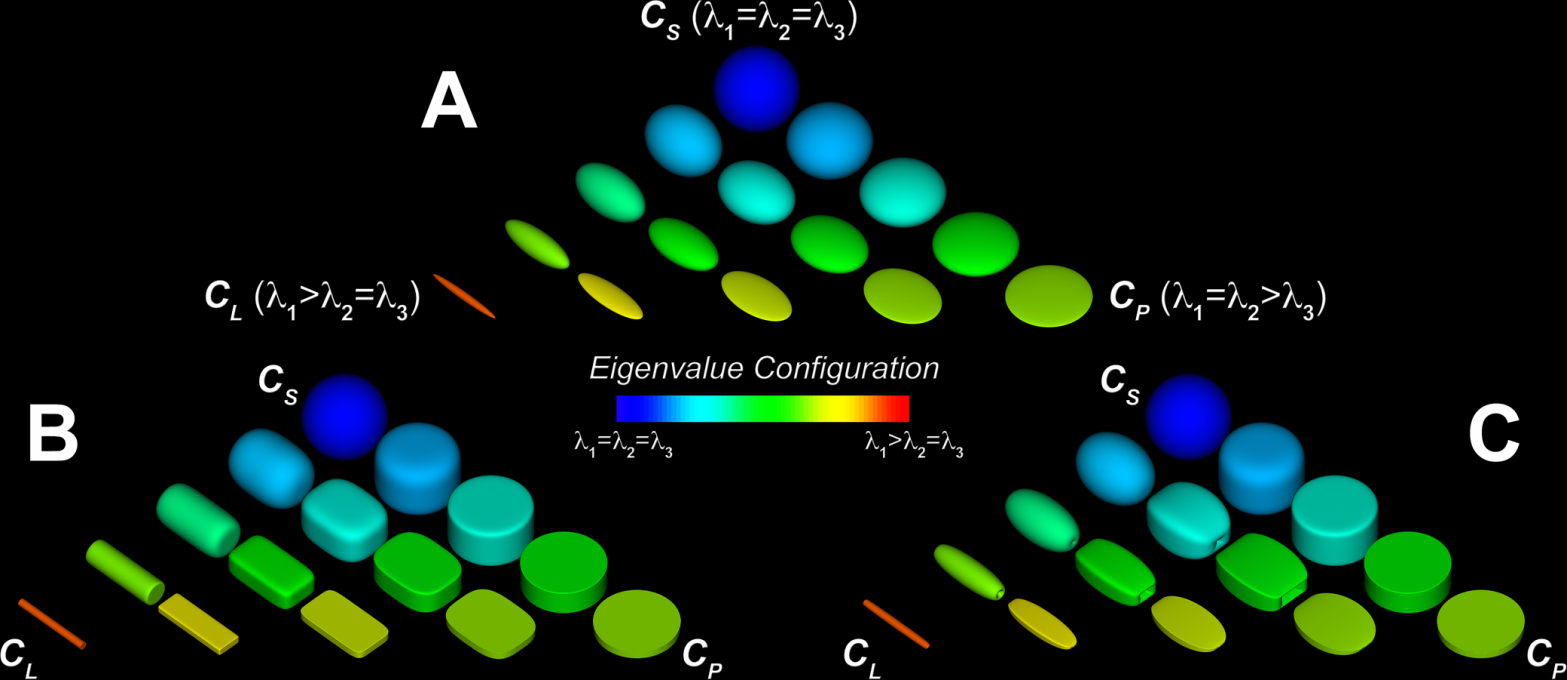
Continua of possible shapes for ellipsoidal, superquadric, and supertoroidal glyphs. Ellipsoidal (A), superquadric (B), and supertoroidal (C) glyphs are functions of geometric shape metrics C_S_, C_P_, and C_L_. Glyph shapes are rendered at 30 degrees with respect to the viewer. The glyph field is color-coded according to eigenvalue configuration. Supertoroids overcome some of the visual ambiguities associated with ellipsoids and superquadrics due to the increase in shape genus.

Fig 1A, 1B and 1C illustrate three representative continua of possible shapes for ellipsoidal, superquadric, and supertoroidal glyphs. The vertices of each triangular continuum represent the extreme values for linear anisotropy (high C_L_), planar anisotropy (high C_P_), and spherical isotropy (high C_S_). Visualization of the ellipsoidal field (Fig 1A) demonstrates that ellipsoids can be an ambiguous tensor representation due to their lack of distinct surface features. It is straightforward to differentiate the extreme cases of isotropy and anisotropy, but full appreciation of the diffusion magnitude and orientation in three dimensions may require several viewing perspectives. Hence, a more effective representation for diffusion tensor visualization and characterization is desirable.

Superquadrics (Fig 1B) provide a blend of different shapes in which the variation in anisotropy mode is described by the sharpness of the glyph’s edges. Superquadrics depend on a free parameter, *γ*, to determine how distinctly eigenvectors are represented [12]. While superquadrics reduce some of the visual ambiguities observed with ellipsoids, the lack of features intrinsic to a genus 0 shape limits their ability to provide an unambiguous representation of diffusion tensor fields.

Supertoroids (Fig 1C) improve upon the superquadrics by employing a genus 1 shape, the toroid, to further eliminate the remaining ambiguities of the conventional genus 0 representations. Supertoroids depend on two free parameters, *γ*_1_ and *γ*_2_, that determine how shape features will change according to different degrees of anisotropy. In addition, supertoroids also indicate the principal orientation of local diffusion, which is aligned with the opening, regardless of the viewing perspective. Finally, variations in the eigenvalue configuration are depicted by changes in the supertoroid through the use of a blend of genus 1 shapes.

### Toroidal indices

While differences in eigenvalue configurations can be intuitively inferred from supertoroidal glyphs, for quantitative analyses, it is more useful to measure changes in diffusion tensor fields by computing scalar indices of diffusivity and anisotropy. In order to define tensor invariants that are independent of the display parameters (*γ*_1_ and *γ*_2_) of the supertoroidal model, we derived a toroidal function where the eigenvalues are the only parameters that modify the toroidal shape.

The toroidal surface *T* is defined as a function of both the azimuthal *θ* ∈ [0, 2*π*] and polar *ϕ* ∈ [0, 2*π*] coordinates as follows:

$$T\left(\theta ,\phi \right)=\left(\begin{array}{c} cos\theta \left(\alpha +\beta cos\phi \right)\\ \text{sin}\theta \left(\alpha +\beta cos\phi \right)\\ \gamma \text{sin}\phi \end{array}\right),$$ (3)

where: *α* = (2*λ*_2_ + *λ*_3_)/4, *β* = *λ*_3_/4, and *γ* = *λ*_1_/2.

This parameterization restricts the shape of the toroid to vary from a tube in regions of high anisotropy to an ordinary torus in those characterized by high isotropy. The toroidal model is parameterized such that *λ*_1_ is the extent along the rotational axis of the toroid, *λ*_2_, the diameter of the central opening, and *λ*_3_/2, the thickness of the toroidal cross-section.

### Diffusivity

Diffusivity is commonly estimated using the trace of the tensor (sum of the eigenvalues) or the mean diffusivity (MD), defined as *MD* = 〈*λ*〉 = (*λ*_1_ + *λ*_2_ + *λ*_3_)/3, which represents the average displacement of a water molecule over the echo time (TE). While MD is a mono-dimensional linear function, diffusion is inherently a three-dimensional phenomenon. The motion of water molecules increases the tridimensional space they occupy over time. This increase in volume depends on the diffusivity of the medium and on the presence of boundaries and is characterized by the eigensystem where the eigenvectors represent the directions of volume increase and the eigenvalues represent their respective magnitude [2]. Because diffusion is a volumetric phenomenon, it is intuitive to use a volumetric scalar parameterized by the eigenvalues to quantify it. Thus, we define the toroidal volume (TV) as an index of diffusivity from the toroidal parameterization in (3):

$$TV=\left(\lambda_{1}\pi /3\right)\left(\lambda_{2}\lambda_{3}+\lambda_{3}{}^{2}/2\right)$$ (4)

**Fig 2:**
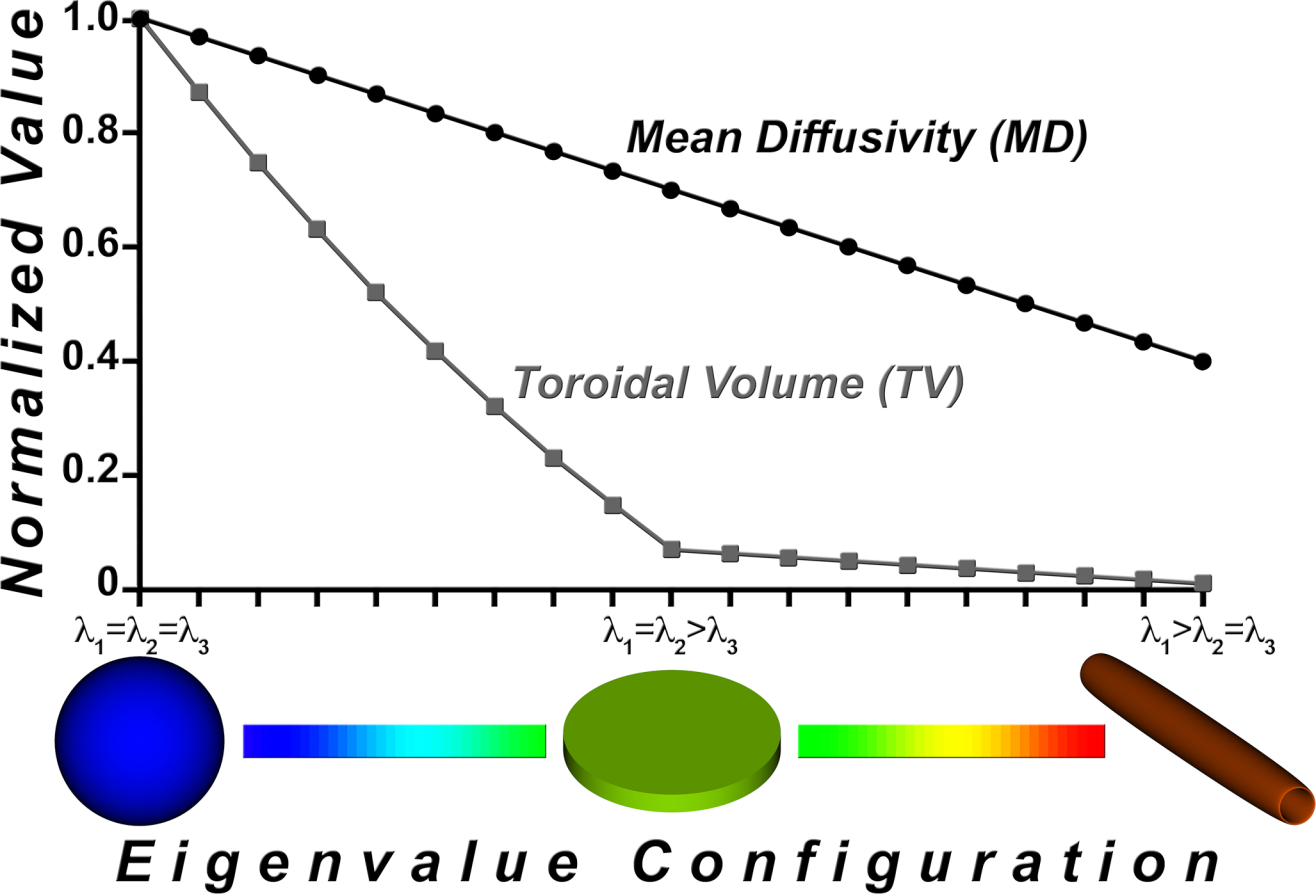
Comparison of the toroidal volume (TV) and mean diffusivity (MD) indices. Diffusivity indices TV and MD, each normalized to [0, 1], and displayed as a function of eigenvalue configuration. MD is a linear function whereas TV exhibits a nonlinear response to the increase in anisotropy. The inflection point in the TV curve profile from isotropy to anisotropy enhances tissue discrimination as compared to MD.

As TV is a function of the product of the three eigenvalues, it is a measure of volume (expressed in mm^6^/s^3^) and is therefore nonlinear (Fig 2).

### Anisotropy

The measure of anisotropy reflects the degree of directional dependence of the physical restriction in the medium during the expansion of water molecules over time [10]. It is generally quantified by the ellipsoidal eccentricity, also known as the fractional anisotropy (FA) index:

$$FA=\sqrt{1/2}\left(\sqrt{{\left(\lambda_{1}-\lambda_{2}\right)}^{2}+{\left(\lambda_{2}-\lambda_{3}\right)}^{2}+{\left(\lambda_{1}-\lambda_{3}\right)}^{2}}/\sqrt{\lambda_{1}{}^{2}+\lambda_{2}{}^{2}+\lambda_{3}{}^{2}}\right)$$ (5)

**Fig 3:**
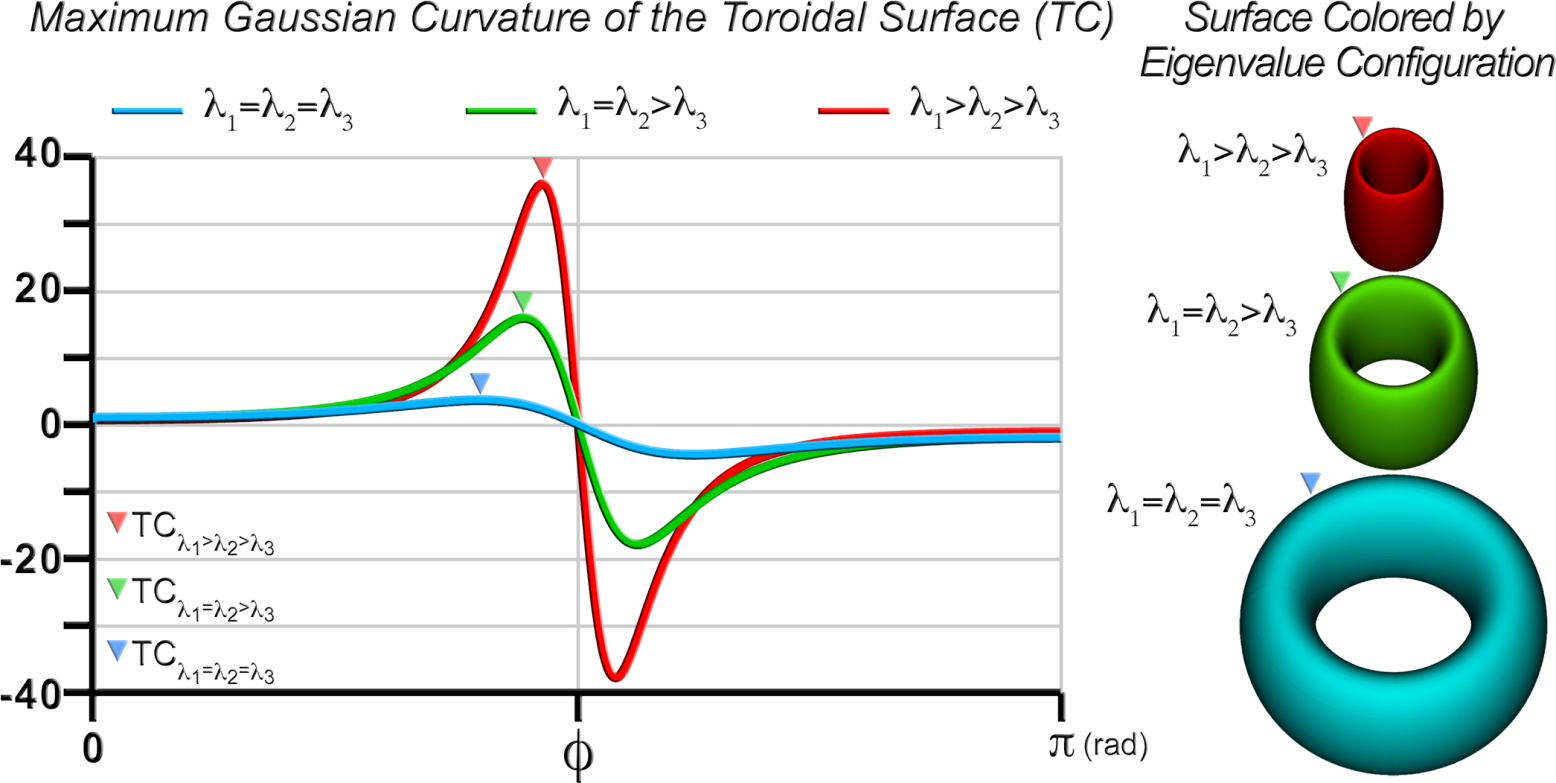
Toroidal curvature (TC) as a function of eigenvalue configuration. Gaussian curvature of the toroidal surface is shown as a function of the polar angle (*ϕ*) in isotropy (blue), transverse isotropy (green), and anisotropy (red). TC is the maximum of the Gaussian curvature values, as indicated at the positive peaks. The corresponding toroidal surfaces and curvature maxima are shown on the right panel.

Using the toroidal model, one can derive a complementary index of anisotropy using the Gaussian curvature of the toroidal surface. According to the toroidal parameterization in (3), the maximal Gaussian curvature values are obtained at the rim of the toroid. As observed in Fig 3, the maximum Gaussian curvature of the toroidal surface (TC) increases monotonically from isotropy (ordinary torus shown in blue) to anisotropy (tube shown in red). TC is defined as:

$$TC=tc\left(\phi_{M}\right)=4\beta^{\prime }\gamma^{\prime \;2}cos\phi_{M}/\left(\left(\alpha^{\prime }+\beta^{\prime }cos\phi_{M}\right){\left[\beta^{\prime \;2}+\gamma^{\prime \;2}+\left(\gamma^{\prime \;2}-\beta^{\prime \;2}\right)cos2\phi_{M}\right]}^{2}\right),$$ (6)

where *α*′ = *α*/*λ*_1_ = (2*λ*_2_ + *λ*_3_)/4*λ*_1_, *β*′ = *β*/*λ*_1_ = *λ*_3_/4*λ*_1_, and *γ*′ = *γ*/*λ*_1_ = 1/2, with $\phi_{M}=\underset{\phi }{\text{argmax}}\{tc(\phi )\},\;\phi \in [0,\pi ]$.

Because TC is a measure of curvature, its range is [0, +∞]. Given that this range is much greater than that of FA, it can better characterize variations in regions of high anisotropy.

### Data analysis and statistics

For each patient dataset containing a GBM, we calculated the diffusion tensor field and the associated scalar maps of diffusivity (MD and TV) and anisotropy (FA and TC). T2-weighted images were used to identify tumor location as well as boundaries between necrotic core and edema. Regions of interest (ROIs) were then selected inside the necrotic core and within its periphery (edema). Additional ROIs were selected at the contralateral side to the lesions, at white (corpus callosum) and gray matter (GM) locations. We sampled the tumor, edema, WM, and GM using ROIs of equal volume (~2500 mm^3^). However, the number of voxels per slice varied as these regions have different geometry in each case. The tumors in these datasets, including necrotic core and edema regions, ranged in size from ~8000 mm^3^ to ~129000 mm^3^. The anatomical locations of the ROIs chosen on the contralateral side to the lesions were carefully selected to be consistent among all datasets and with respect to the location of the GBM. We computed the average value of each index for all individual ROIs. The presence of variation in the diffusion indices across ROIs was assessed non-parametrically using the Friedman test. When variation across regions occurred, post-hoc sign tests were conducted to identify which regions exhibited significantly different values within the ROI. Differences in DTI indices between tumor, edema, GM, and WM were statistically assessed for the toroid- and ellipsoid-based indices. DTI indices were also calculated from datasets of healthy volunteers to be used for visual comparison.

## Results

### Supertoroidal visualization

**Fig 4:**
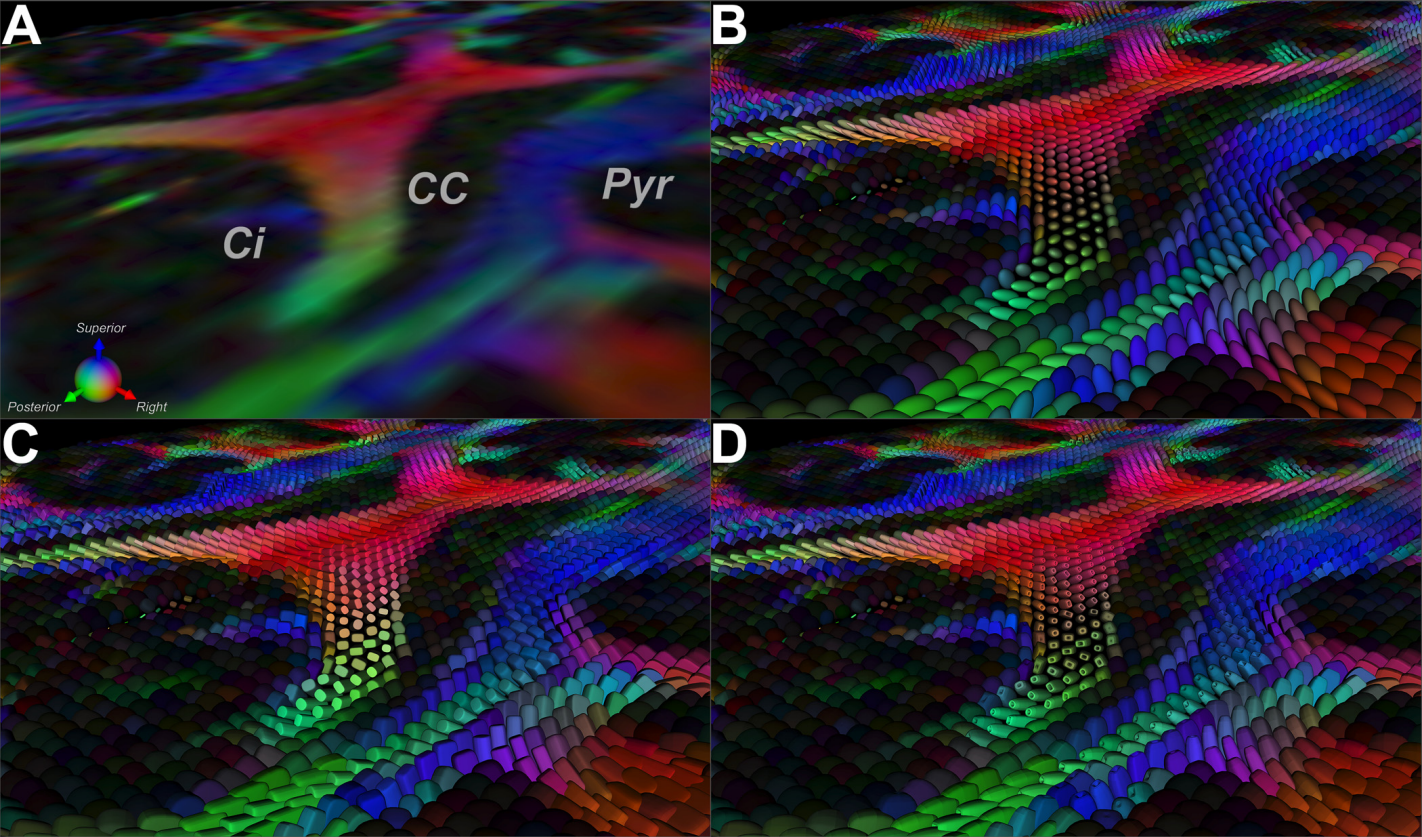
Diffusion tensor representations of a normal brain near the splenium. FA map near the splenium in a normal brain color-coded by the orientation of the primary eigenvector (A). Rendering of this region using ellipsoidal (B), superquadric (C), and supertoroidal (D) glyphs using the same orientation encoding. Supertoroidal glyphs enhance the visualization of local structure and orientation due to the increase in the shape genus.

Supertoroidal glyph renderings of DTI fields may be used to characterize regions of tissue structure in the brain. Fig 4 depicts a mid-axial projection of a normal brain near the splenium. Three major WM tracts can be visually identified in this region: the cingulum (Ci), the corpus callosum (CC), and the pyramidal (Pyr) tracts. The FA map color-coded by the orientation of the primary eigenvector was used to visually determine the location and orientation of these tracts [30]. Fig 4B, 4C and 4D depict the ellipsoidal, superquadric, and supertoroidal representations of this region. Note that the principal orientation of the cingulum and part of the pyramidal tracts are more easily identified because the glyphs are colored according to their orientation (Fig 4C and 4D). The supertoroidal representation renders both isotropic and anisotropic cases effectively since the parameterization allows the glyph to collapse into a genus 0 shape in isotropic regions or remain a genus 1 shape in those that are anisotropic. Therefore, the number of required perspectives to fully comprehend the tensor field is reduced as compared to other representations.

### Toroidal indices

**Fig 5:**
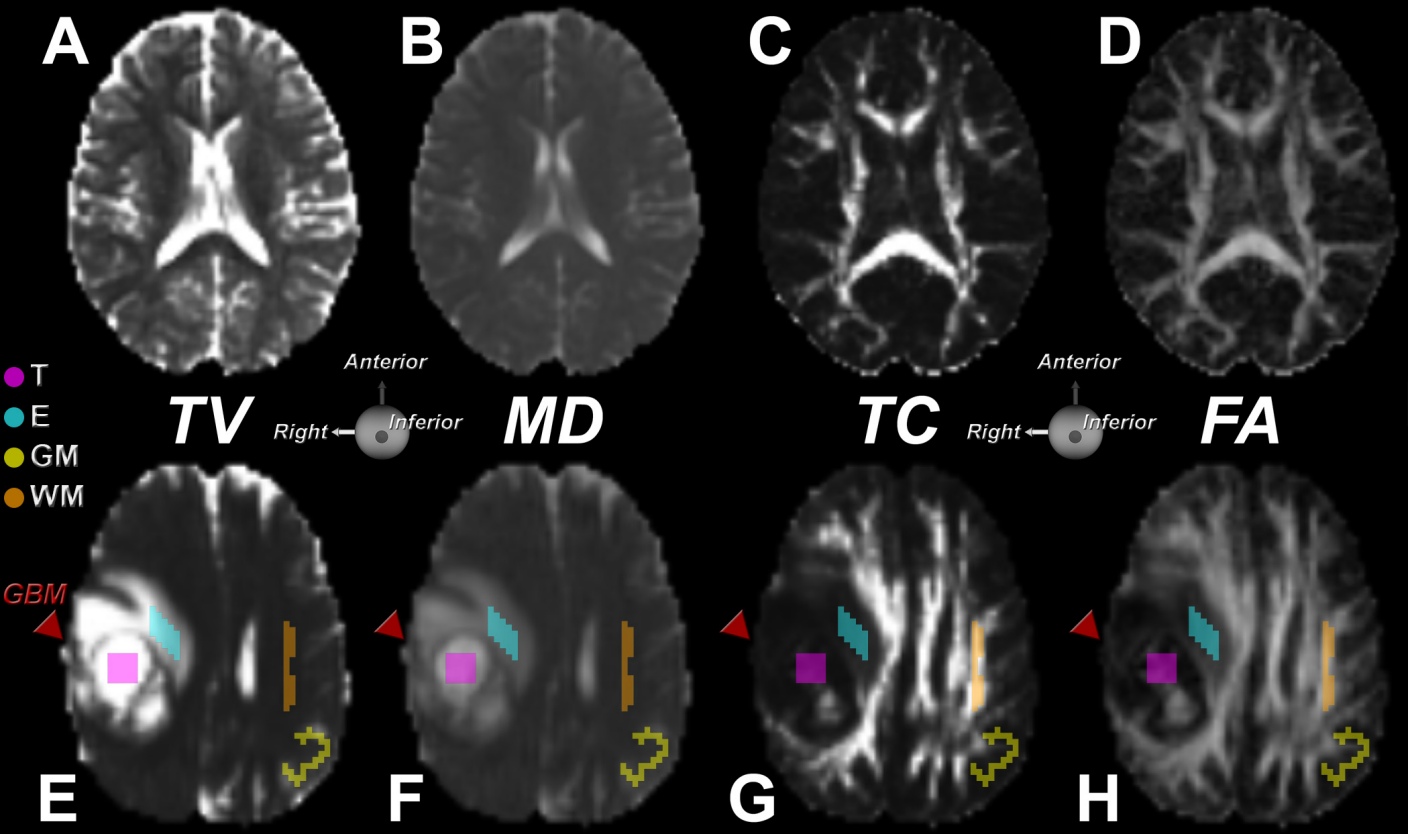
Comparison between toroidal and traditional diffusion tensor maps. Top row shows TV (A), MD (B), TC (C), and FA (D) maps of a mid-axial slice of the brain of a healthy volunteer. In the TV map of the normal brain, a distinction can be made between white matter (WM) and gray matter. The anisotropy coefficient TC displays the WM structure with detail equivalent to the FA map. Bottom row shows TV (E), MD (F), TC (G), and FA (H) maps of a mid-axial slice of a brain containing a GBM (red arrow). The ROIs in the tumor, edema, grey and white matter regions are indicated. The TV map (E) reveals WM information, which is less apparent in the MD map (F). The anisotropy coefficient TC displays some vestiges of WM in the tumor region also evident in the FA map.

Fig 5 illustrates TV, MD, TC, and FA maps for a normal brain and one containing a GBM. In the dataset with a GBM, 2D profiles of the 3D ROIs defined in each region (T, E, GM, and WM) are displayed. Due to the nonlinearity of TV, additional anatomic structural differences can be identified (Fig 5A), where differences in GM and WM are enhanced. In the MD map (Fig 5B), one can easily spot the cerebrospinal fluid-filled regions, such as the ventricles, but the distinction between GM and WM is less clear. By visual inspection, TV appears to be more sensitive to the transition in tissue properties than MD. It can be observed from Fig 5C and 5D that both TC and FA maps convey anisotropy information. The TC map emphasizes regions with high anisotropy, such as the major WM tracts, but does not highlight the smaller fascicles, which are more prominent in the FA map.

Fig 5E depicts a TV map that illustrates a GBM involving the GM and WM of a patient. Both TV and MD (Fig 5F) display high intensities in the necrotic core, with increased values in the adjacent regions as compared to unaffected tissue [31]. However, the TV map displays higher contrast than the MD map, revealing subtle variations in tissue structure that are not easily identified by MD, such as at the boundary of the tumor.

Both the TC and FA (Fig 5G and 5H) maps confirm the degradation in structure of several WM tracts. The mass effect that the tumor exerts on the adjacent WM is also evident in the TC map. While FA and TC maps reveal remnants of normal structure inside the tumor region, the TC map shows lower contrast in this region. Whereas FA may overestimate the degree of anisotropy globally [26], TC tends to be more selective and displays a high degree of anisotropy only for highly linear structures, such as WM. The TC map of the normal brain demonstrates this effect with increased contrast between adjacent tissue structures.

### Supertoroidal representation of a GBM

**Fig 6:**
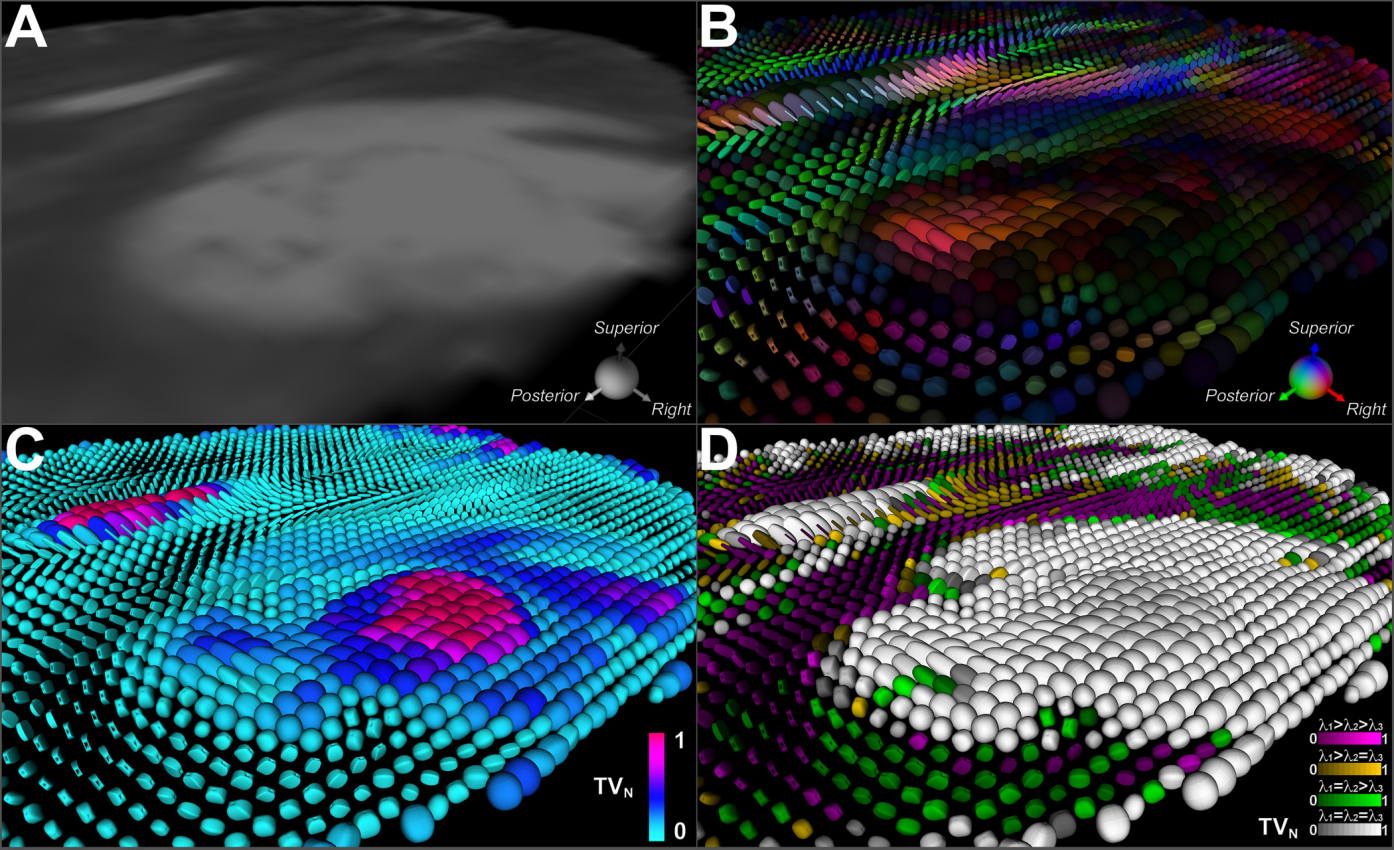
Supertoroidal field of a brain with a GBM color-coded with TV and eigenvalue configuration. (A) T2-weighted image of a mid-level axial cross-section of a brain with a GBM. (B) Supertoroidal glyph field color-coded by the orientation of the primary eigenvector and weighted by FA. (C) Supertoroidal glyph field color-coded with normalized TV index using a hot-to-cold color map, revealing the necrotic core and surrounding edema. (D) Supertoroidal glyphs encoded by the eigenvalue configuration with color saturation modulated by TV. These schemes (C, D) provide visual cues that enhance the identification of tissue architectural changes, as seen in the tumor-infiltrated tissue.

Fig 6A depicts a mid-level axial cross-section of a T2-weighted image of a patient with a GBM. This projection is the same as that illustrated in Fig 4. Fig 6B displays the supertoroidal glyph field color-coded by the orientation of the primary eigenvector and weighted by FA. Fig 6C displays the supertoroidal field overlaid with TV normalized to [0,1] depicted using a hot-to-cold color map. One can observe two predominant distributions of TV in the tumor region, characterizing the necrotic core and surrounding edema. A supplementary color-based classification scheme is introduced to locally distinguish each eigenvalue configuration case as follows:*λ*_1_ > *λ*_2_ > *λ*_3_, shown in purple; *λ*_1_ > *λ*_2_ = *λ*_3_, shown in orange; *λ*_1_ = *λ*_2_ > *λ*_3_, shown in green, and *λ*_1_ = *λ*_2_ = *λ*_3_, shown in gray. In addition, the brightness component of each color is modulated according to TV. This color-coding scheme provides a visual cue with which to identify architectural changes in the tissue (Fig 6D). Note that fiber orientation remains intact in the transition from normal tissue to edema, but degrades progressively as it reaches the necrotic core. Much of the tumor region is isotropic, as evidenced by the glyphs rendered using the gray scheme representing tumor infiltrated cells, while surrounding regions still preserve some of the original structure, which can be seen by the orange and purple glyph clusters. This is in agreement with findings by Lazar *et al*., which suggest that normal WM voxels fall in the *λ*_1_ > *λ*_2_ > *λ*_3_ configuration [32].

### Statistical analysis

**Fig 7:**
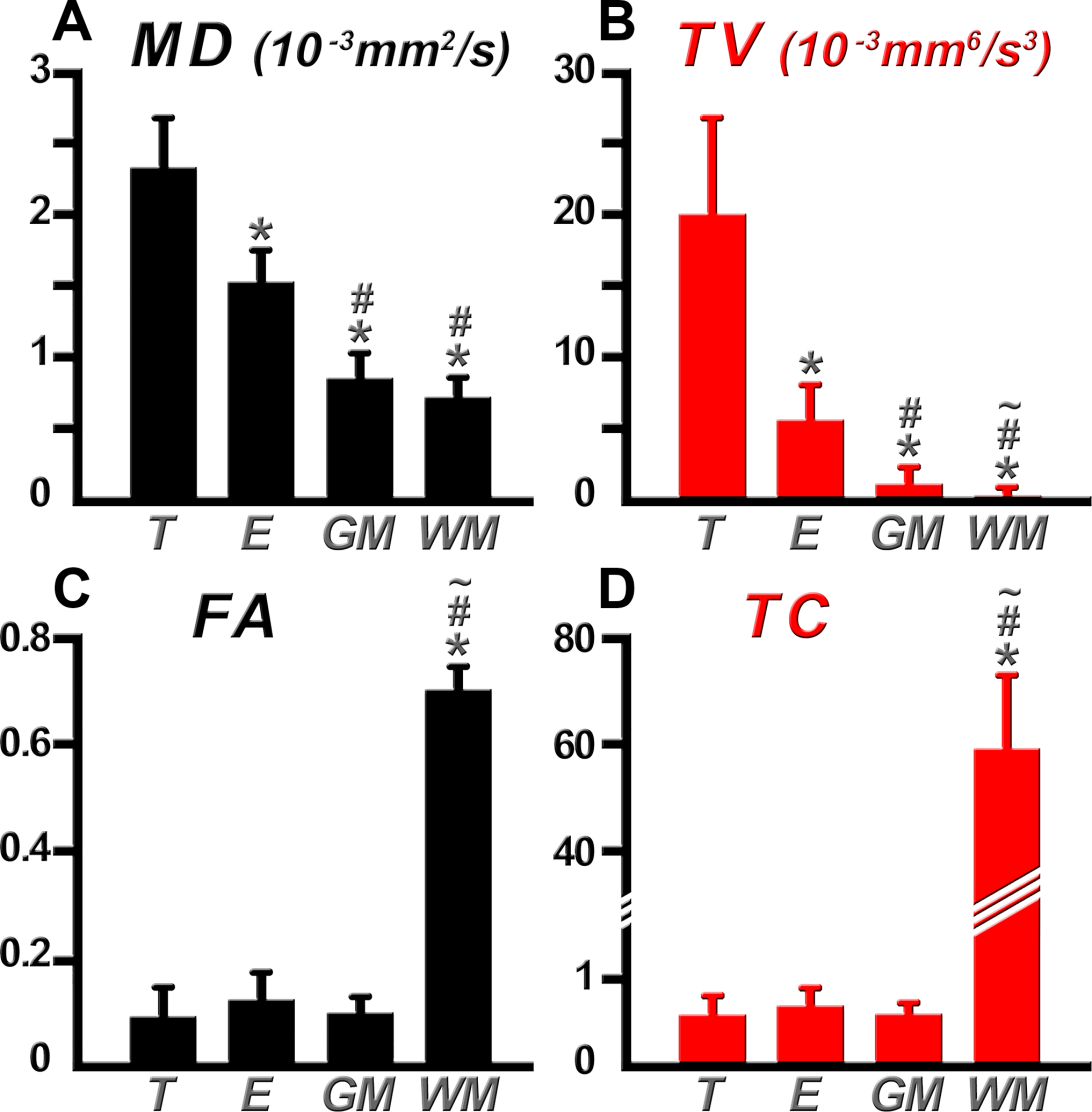
Comparison of MD, TV, FA, and TC within different regions of interest. Bar plots representing MD (A), TV (B), FA (C), and TC (D) values within tumor (T), edema (E), gray matter (GM), and white matter (WM). Significant differences (p<0.05) are denoted between the T region (*), E region (#), and GM region (~) with respect to other ROIs. Discrimination of more tissue types was possible with TV than with MD owing to the nonlinear response of TV. The greater range of TC improves discrimination between degrees of anisotropy within the same WM structure and provides complementary information to that of FA.

Differences in DTI indices between tumor, edema, GM, and WM regions in patients with GBM were statistically assessed for the toroid- and ellipsoid-based indices. Variations across regions revealed differences in MD (p = 0.0007), TV (p = 0.0004), FA (p = 0.0067), and TC (p = 0.0089), using the Friedman test. Follow-up tests were conducted using the sign test to identify the regions with significantly different diffusion parameters (p<0.05). Significant differences were found in MD between tumor and edema, tumor and GM, as well as tumor and WM (Fig 7A). MD was also able to distinguish edema from GM and edema from WM, but was not able to differentiate GM from WM (p = 0.0625). This is a consequence of the reduced contrast observed between these regions in the MD maps. TV, like MD, was able to distinguish tumor from edema and all other regions (Fig 7B). In contrast to MD, TV was able to statistically discriminate GM from WM (p = 0.0313).

Both coefficients of anisotropy, FA (Fig 7C) and TC (Fig 7D), were able to differentiate tumor from WM but could not distinguish tumor from GM or edema. A significant difference between edema and WM was also found. As is typically observed with FA and TC maps, WM and GM regions are easily identified by their different intensity distributions. This difference was statistically verified in this analysis. The greater range of TC improves discrimination between degrees of anisotropy within the same WM structure. The results indicate that both FA and TC provide complementary information on anisotropy and can help in tissue discrimination.

## Discussion

DTI has recently become a powerful tool in neurology and neuro-oncology leading to an improvement in both diagnosis and prognosis of several types of brain neoplasms as well as in neurosurgical guidance during brain tumor surgery [4–9]. Successful characterization of biological tissue using DTI, however, relies on effective visualization and analysis methods. While ellipsoids and superquadrics have improved upon the ability to illustrate local differences and continuous features in tensor fields, one drawback is their limited ability to indicate the orientation of the primary eigenvector depending on the viewing perspective. In this work, we describe the supertoroidal representation of the dyadic diffusion tensor. A fundamental feature of the supertoroidal model is the use of a genus 1 shape, which provides a clear indication of the principal orientation of the local diffusion, regardless of scene orientation. In addition, the supertoroidal shape is a function of two free parameters (*γ*_1_ and *γ*_2_), which modulate the sharpness of the supertoroid in the medium and minor diffusion directions and the shape along its orthogonal axis, respectively. The choice of *γ*_1_ and *γ*_2_ depends on the properties of the biological tissue under investigation. For instance, these two parameters could be tuned to enhance visual discrimination of different tissues, depending on the application. Thus, as a result of the increase in surface genus and the variability in shape as a function of *γ*_1_ and *γ*_2_, the supertoroidal model improves the interpretation of DTI images of the brain in normal and pathological conditions.

**Fig 8:**
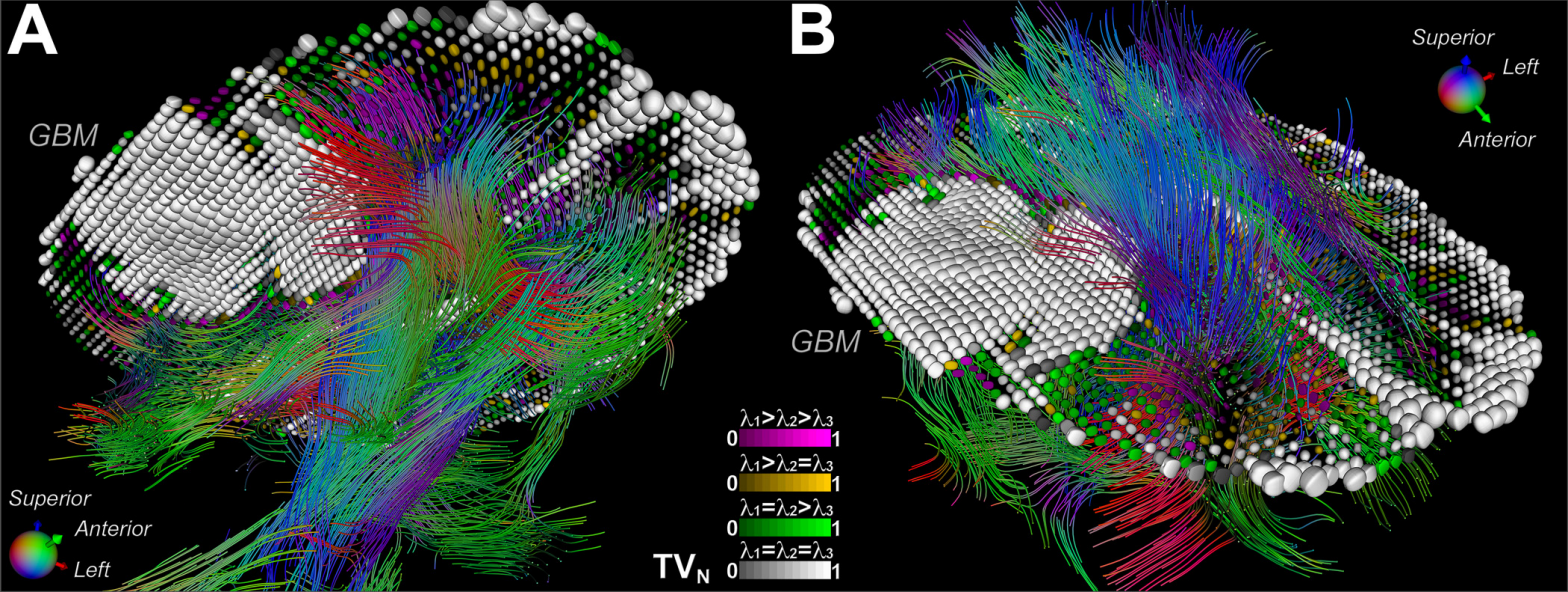
Mass effect due to a GBM depicted by the integration of tractography with supertoroids. Tractography (with conventional orientation encoding) of the white matter in the presence of a GBM, augmented with supertoroidal glyphs color-coded by eigenvalue configuration (A, B). This demonstrates the mass effect exerted by the GBM, in which surrounding white matter tracts are pushed away from their normal trajectories as a result of tumor growth.

We also describe diffusivity (TV) and anisotropy (TC) indices derived from the toroidal function. An evaluation of these indices using human brain DTI (Fig 5) showed that these toroid-based invariants contain complementary information to the traditional MD and FA indices. The nonlinear property of TV enhances edge features in TV maps, which may lead to improved tissue discrimination as demonstrated by the refined boundaries between WM and GM (Figs 4 and 7). The toroid-based indices corroborate observed regional variations in diffusivity and anisotropy as measured by the standard diffusion invariants [23]. The combination of both diffusivity indices may allow for a more accurate identification of boundaries between cerebral neoplasms and surrounding structures. As a consequence, the resultant subcortical landmarks may be useful for optimization of surgical planning and intraoperative image guidance during brain tumor surgery in eloquent areas [33, 34]. In addition, integration of tractography with supertoroids further reveals the disruption to the local tissue environment by cerebral neoplasms. Fig 8 demonstrates one such integration, depicting the mass effect due to a GBM, in which surrounding WM tracts are pushed away from their normal trajectories as a result of tumor growth. Integration of the supertoroidal model and tractography enhances the 3D structure of the tumor and the surrounding parenchyma. This also provides complementary information about the trajectory of potentially-compromised WM fibers, which can be valuable in neurosurgical planning and is an active and challenging area of research [35].

Limitations of this study include the relatively small sample size and the use of diffusion tensor imaging without other modalities (e.g. T1, FLAIR, fMRI). Our study focus on full-tensor indices of diffusivity and anisotropy rather than selective combinations of eigenvalues, adding to a growing body of work suggesting the utility of DTI in the assessment of cerebral lesions. It has been demonstrated that early structural changes can be initially detected by diffusivity indices and subsequent architectural alterations are revealed by anisotropy coefficients [36], which suggests that TV may complement MD in the detection of early lesions, whereas TC and FA may contribute to the characterization of subsequent architectural alterations. For example, the progressive breakdown of the myelin sheath and cell membrane could be measured by both MD and TV. The toroidal model has the potential to improve diagnostic accuracy and outcome prediction provided by traditional diffusion indices. However, further clinical studies are required to evaluate the sensitivity and specificity of both TV and TC in the detection and delineation of cerebral pathology.

## Conclusions

The supertoroidal model was used to explore macrostructural features of the brain from DTI images, in both normal and pathological states. The proposed approach can be easily extended to the visualization and analysis of other organ systems and also tensor fields arising from non-biological material [37]. Such analyses could be used to explore the relationship between material properties and the diffusion process. In conclusion, the supertoroidal representation simultaneously offers effective tensor visualization as well as quantitative scalar maps and may provide meaningful information in terms of diagnosis, prognosis, and therapeutic management of brain lesions.
